# Recurrent insertion and duplication generate networks of transposable element sequences in the *Drosophila melanogaster *genome

**DOI:** 10.1186/gb-2006-7-11-r112

**Published:** 2006-11-29

**Authors:** Casey M Bergman, Hadi Quesneville, Dominique Anxolabéhère, Michael Ashburner

**Affiliations:** 1 Department of Genetics, University of Cambridge, Cambridge CB2 3EH, UK; 2 Faculty of Life Sciences, University of Manchester, Manchester M13 9PT, UK; 3 Laboratoire de Bioinformatique et Génomique, Institut Jacques Monod, place Jussieu, 75251 Paris cedex 05, France; 4 Laboratoire Dynamique du Génome et Évolution, Institut Jacques Monod, place Jussieu, 75251 Paris cedex 05, France

## Abstract

**Background:**

The recent availability of genome sequences has provided unparalleled insights into the broad-scale patterns of transposable element (TE) sequences in eukaryotic genomes. Nevertheless, the difficulties that TEs pose for genome assembly and annotation have prevented detailed, quantitative inferences about the contribution of TEs to genomes sequences.

**Results:**

Using a high-resolution annotation of TEs in Release 4 genome sequence, we revise estimates of TE abundance in *Drosophila melanogaster*. We show that TEs are non-randomly distributed within regions of high and low TE abundance, and that pericentromeric regions with high TE abundance are mosaics of distinct regions of extreme and normal TE density. Comparative analysis revealed that this punctate pattern evolves jointly by transposition and duplication, but not by inversion of TE-rich regions from unsequenced heterochromatin. Analysis of genome-wide patterns of TE nesting revealed a 'nesting network' that includes virtually all of the known TE families in the genome. Numerous directed cycles exist among TE families in the nesting network, implying concurrent or overlapping periods of transpositional activity.

**Conclusion:**

Rapid restructuring of the genomic landscape by transposition and duplication has recently added hundreds of kilobases of TE sequence to pericentromeric regions in *D. melanogaster*. These events create ragged transitions between unique and repetitive sequences in the zone between euchromatic and beta-heterochromatic regions. Complex relationships of TE nesting in beta-heterochromatic regions raise the possibility of a co-suppression network that may act as a global surveillance system against the majority of TE families in *D. melanogaster*.

## Abstract 2

An analysis of high-resolution transposable element annotations in Drosophila melanogaster suggests the existence of a global surveillance system against the majority of transposable elements families in the fly.

## Background

Nearly all eukaryotic genomes contain a substantial fraction of middle repetitive, transposable element (TE) sequences interspersed with the unique sequences encoding genes and *cis*-regulatory elements. The broad-scale patterns of TE abundance and distribution in various model organisms have become increasingly well-understood with the recent availability of essentially complete genome sequences (for example, [1-4]). Despite these general advances, however, a detailed understanding of the evolutionary forces that control the abundance and distribution of TEs remains elusive, owing in part to the dynamic nature of this component of the genome as well as to the inherent problems that TE sequences present for genome assembly and annotation.

As with all unfinished whole-genome shotgun assemblies, uncertainty in the assembly of repetitive DNA in the first two releases of the *Drosophila melanogaster *genome sequence posed difficulties for analysis of TE sequences [5-8]. The improved assembly of repetitive regions in the *D. melanogaster *Release 3 genome sequence presented the first opportunity to study TEs in a finished whole genome shotgun sequence [2,9], revealing the true challenge that these sequences pose for their systematic annotation [10,11]. With further improvements in the Release 4 genome sequence made possible by the efforts of the Berkeley *Drosophila *Genome Project [12] (especially in regions of high TE density where several gaps have been completed), we are now in a position to establish more stable trends in TE abundance for *D. melanogaster*. In addition to having access to improved genome sequence data, we have recently developed an improved TE annotation pipeline that uses the combined evidence of multiple computational methods to predict 'TE models' in genome sequences [10]. We have shown that this pipeline identifies a large number of predicted TEs that were omitted from the Release 3 genome annotations, and subsequently applied this system to the *D. melanogaster *Release 4 sequence [10]. Here we analyze the results of this effort in detail, which allows an extremely high-resolution view of the structure and location of TEs in one of the highest quality metazoan genome sequences currently available.

We first revised baseline estimates of the TE abundance in the *Drosophila *genome sequence, based on the fact that TEs show a strikingly non-random distribution across the genome. We then used this baseline to identify specific regions of extremely high TE density in the genome sequence. This analysis showed that regions of the genome broadly known to have high TE abundance, such as pericentromeric regions and the fourth chromosome, are in fact often characterized by distinctly localized regions of extremely high TE density interrupted by regions of lower TE density. Comparative sequence analysis showed that this punctate pattern is unlikely to have arisen in the *D. melanogaster *genome by inversion of TE-rich heterochromatic sequences, but can evolve *in situ *by the joint action of recurrent transposition and duplication. Finally, we analyzed in detail the patterns of TE nesting in the genome sequence, taking advantage of the improved joining of fragments from the same TE insertion event in our new annotation. We framed the process of TE nesting as a directed graph and borrowed techniques from network analysis to study genome-wide patterns of TE nesting. This work demonstrates the added value of high-resolution annotations for understanding how TEs impact genome organization and evolution, and preludes the interpretation of TE-rich heterochromatic regions currently being sequenced by the *Drosophila *Heterochromatin Genome Project [13].

## Results

### Abundance and distribution of TEs in the Release 4 genome sequence

Using a recently completed combined-evidence annotation of the Release 4 genome sequence [10], we revised estimates of the overall abundance of TE sequences in *D. melanogaster *(Table 1) from those based on the Release 3 sequence [2]. Excluding foreign elements based on query sequences from other species (see Materials and methods), the estimated number of TEs in the *D. melanogaster *Release 4 genome sequence (*n *= 5,390) is over three-fold higher than in Release 3 (*n *= 1,572). In contrast, the amount of sequence annotated as TE increased by only approximately 44% in Release 4 (6.51 Mb, 5.50% of genome) relative to Release 3 (4.51 Mb, 3.86% of genome). (We note that the proportion of the Release 4 genome estimated here as TE is calculated as the sum of non-redundant annotation spans including unique sequences inserted into TEs; this procedure differs slightly from our previous estimates for Release 4, which only included sequences strictly homologous to TE query sequences [10].) The discrepant changes in these two metrics of TE abundance across releases results from the fact that almost all new TEs in Release 4 are either small fragments and/or annotations of the highly abundant but degenerated *INE-1 *element (also known as *DINE-1 *or DNAREP1_DM) [14], a family that was omitted from the Release 3 annotation. The inclusion of these new small fragments is also reflected in the fact that the proportion of TEs estimated to be full-length (defined as ± 3% of the canonical element including the length of inserted sequences) has declined from 30.5% in Release 3 to 9.83% in Release 4. The number of TEs involved in nests (*n *= 785) has more than doubled in Release 4 relative to Release 3 because of newly annotated sequences and improved joining of TE fragments belonging to the same insertion, although the estimated proportion of TEs involved in nests (14.6%) in Release 4 has decreased relative to Release 3 as a consequence of the increased total number of TEs annotated.

**Figure 1:**
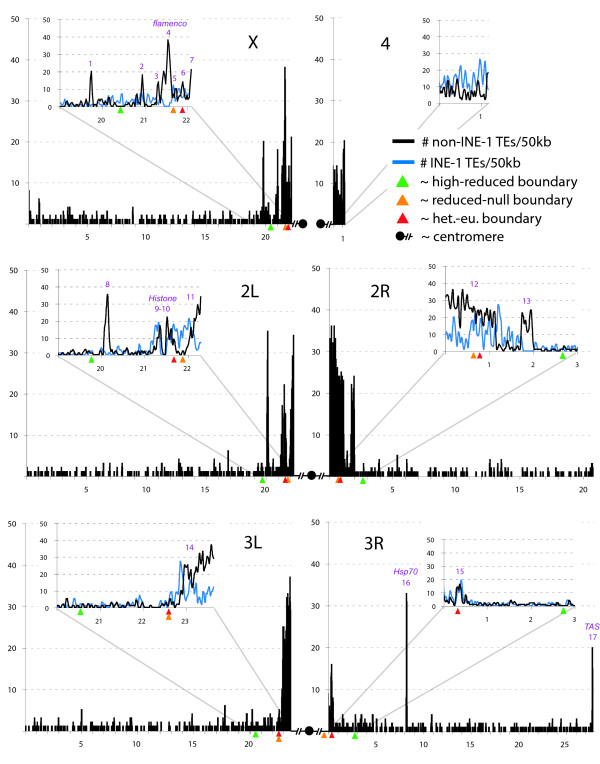
Distribution of TEs along the *D. melanogaster *Release 4 chromosome arms. Numbers of TEs per 50 Kb window are plotted as a function of position along a chromosome arm. Abundance for all families excluding the *INE-1 *is shown in black for the main and inset panels, and in blue for the *INE-1 *family in inset panels. Positions of the cytologically estimated boundaries between euchromatin and heterochromatin in pericentromeric regions are shown as red triangles. Positions of genetically estimated boundaries between high and reduced recombination, and between reduced and null recombination, in pericentromeric regions are shown as green and orange triangles respectively. Filled circles indicate centromeric regions that are currently not included in the Release 4 genome sequence. HDRs on the major chromosome arms are numbered in purple.

The major patterns of TE abundance identified in previous releases of the *D. melanogaster *genome sequence [2,7,8,15,16] are also observed in Release 4, suggesting that these trends are stable features of the *D. melanogaster *genomic landscape. As shown in Figure 1, both the pericentromeric regions of the major chromosome arms and the entirety of chromosome 4 have higher densities of TE insertions, relative to non-pericentromeric regions [2,7,15]. Densities over the non-pericentromeric regions are roughly equal, with no general increase in TE density in telomeric regions (Figure 1) [7,15], excluding TEs that are directly involved in telomere structure/function or in the subtelomeric arrays (see below). There is no general decrease in the abundance of TEs on the X chromosome [2,15], as expected if TE insertions generate deleterious recessive mutations [17]. Long terminal repeat (LTR) retrotransposons occupy the greatest proportion of the genome sequence (3.29%), as has been observed previously [2,7], but the current annotation reveals that the *INE-1 *family is the most numerous category of TEs (*n *= 2,238) in the *D. melanogaster *genome [16]. (We note that throughout this work, non-LTR retrotransposon is abbreviated as 'non-LTR', which is referred to as *LINE*-like in [2,7].) *INE-1 *has previously been suggested to be a retrotransposon on the basis of homology to the *D. virilis Penelope *element [16]; however, we found that this reported homology between *Penelope *and *INE-1 *is spurious and restricted to flanking sequences in GenBank:U49102 (see also [18]). From the percent genome sequence occupied, our analysis indicates that *INE-1 *distribution most closely fits the terminal inverted repeat (TIR) transposon class of TEs (Table 1), supporting the conclusion that *INE-1 *is a TIR element based on structural features of an improved consensus sequence [19].

This set of 5,390 TEs defined 4,684 TE-free regions (TFRs) [20] in the Release 4 genome sequence; 94.5% (111.9 Mb of 118.4 Mb) of the Release 4 genome sequence can be found in TFRs, with 89.8% (106.2 Mb) and 56.1% (66.4 Mb) of the genome found in TFRs of greater than 10 Kb (*n *= 1,393) and 100 Kb (*n *= 357), respectively. The longest TFR in *D. melanogaster *is 855,890 base-pairs (bp) in length on chromosome 2R from 14,374,883-15,230,772, contains 106 genes, and is over 10 times longer than the longest TFR in the human genome [20]. The mean TFR length of 23,878 bp is consistent with the genome-wide minimum estimate of the distance between middle-repetitive interspersed repeats (>13 Kb) based on reassociation kinetics [21]; however, the median TFR length of 1,992 bp is much smaller. The distribution of TFR lengths departs significantly from an exponential distribution parameterized on this mean length using an adjusted Kolmogorov-Smirnov test (D = 0.4513, p < 0.001), which is based on the maximal difference between observed and expected cumulative distributions and accounts for the fact that the rate parameter for the exponential distribution has been estimated from the data [22]. Similar results are obtained if the rate parameter for the exponential is calculated from the number of TE insertions divided by the total length of TFRs (as in [20]), both including (adjusted Kolmogorov-Smirnov test, D = 0.4719, p < 0.001) or excluding (adjusted Kolmogorov-Smirnov test, D = 0.4456, p < 0.001) TEs nested in other TEs. These results are not simply a result of a high density in pericentromeric regions (see below) and demonstrate that the location of TEs is non-randomly distributed at the level of the complete *D. melanogaster *genome sequence, confirming previous results [7,8,15]. We note that TFRs in the *D. melanogaster *genome are likely to vary among individuals since most TE insertions are not fixed in the species [23]; however, these results should be representative of other strains to the extent that the TE composition of the genome sequence reflects general properties of the species [2].

### Pericentromeric regions, non-pericentromeric regions and the fourth chromosome differ drastically in TE content

Since non-random distribution of TEs can lead to greater than one order of magnitude differences in TE abundance in pericentromeric and non-pericentromeric regions [2,7,8,15,24], overall genome-wide summary statistics do not accurately reflect TE abundance for any region of the genome sequence. To account for this heterogeneity, we attempted to partition the major chromosome arms into regions of high (pericentromeric) and low (non-pericentromeric) TE density using an independent criterion that is not based on TE content. Our primary goal here was to estimate the TE content in non-pericentromeric regions of the genome as accurately as possible, to understand baseline levels of TE abundance throughout the majority of the genome. Initially we investigated using a partition based on the cytologically defined boundaries between euchromatin and β-heterochromatin estimated in Hoskins *et al*. [25]. As shown in Figure 1 (red triangles), the cytologically defined limits of the euchromatin/β-heterochromatin boundaries correspond almost exactly to the most distal pericentromeric region of high TE density on chromosome arms 3L and 3R. However, on chromosome arms 2L, 2R and X the most distal pericentromeric regions of extreme TE density are up to 2 Mb from the estimated euchromatin/β-heterochromatin boundary. Thus, using this cytological criterion to partition the genome into regions of high and low TE density still leads to an over-estimate of the true TE abundance for the majority of the genome.

We next evaluated whether genetically defined regions of different recombination rates estimated by Charlesworth [26] could partition the genome into high and low TE density regions. For all chromosome arms (excluding the fourth chromosome), we found that the estimated boundaries between 'reduced' and 'null' (that is, very low) recombination rates in pericentromeric regions (Figure 1, orange triangles) were located extremely close to the cytologically defined boundaries between euchromatin and β-heterochromatin. Thus, the same tendency to bias estimates of TE abundance exists if the boundary between reduced and null recombination rates is used to partition the genome as for the cytological criterion above. In contrast, the estimated transitions between 'high' and 'reduced' recombination rates in pericentromeric regions (Figure 1, green triangles) are approximately 1 to 2 Mb distal to estimated euchromatin/β-heterochromatin boundaries for all major chromosome arms. Virtually all regions with high TE density were included in the 11% of the genome sequence labeled under this definition as 'pericentromeric' (Figure 1), and, therefore, this partition was used to estimate TE abundance in different regions of *D. melanogaster *genome. Because our aim was to estimate the TE content in non-pericentromeric regions as a baseline to identify regions of extremely high TE content elsewhere in the genome, the inclusion of some low TE content regions in pericentromeric regions on chromosome arms 3L and 3R using this partition should not bias estimates of the background TE abundance throughout the euchromatin.

#### Non-pericentromeric regions

A 'typical' region of the *D. melanogaster *Release 4 genome sequence (that is, the 88% of the genome in non-pericentromeric, high recombination regions on the major chromosome arms) contains approximately 3.32% TE sequences, with an average of 16.9 TEs per Mb (Table 1). Previous estimates based on Release 1 and 2 are not meaningful because of assembly errors [7,15], and those based on Releases 3 and 4 were computed across the entire genome [2,10], thus the current figures represent the first unbiased estimates of TE content for the majority of the *D. melanogaster *genome sequence. As observed in previous releases of the *D. melanogaster *genome sequence [2,7], the rank order of abundance of major TE classes in non-pericentromeric regions is: LTR elements (2.42%, 4.96/Mb) > non-LTR elements (0.62%, 3.24/Mb) > TIR elements (0.15%, 2.06/Mb). *INE-1 *elements account for only 0.10% of a typical region of the *D. melanogaster *genome, but contribute 6.36 TEs/Mb. Approximately 20.5% of the TEs in non-pericentromeric regions are estimated to be full-length (± 3% of the canonical element including the length of inserted sequences), although this value will undoubtedly change with different definitions of what constitutes a full-length element. Virtually every TE in non-pericentromeric regions exists as an individual insertion, with only 6.41% involved in nests of TEs inserted into other TEs. The majority of TE families (97/121, 80.2%) present in the genome sequence have copies in non-pericentromeric regions.

#### Pericentromeric regions

In stark contrast, the 11% of the genome sequence in pericentromeric, low-recombination regions on major chromosome arms contains 57.5% (*n *= 3,101) of the 5,390 TEs annotated and 42.7% (2.78 Mb) of the 6.51 Mb of sequence annotated as TE. On average, pericentromeric regions are composed of 20.9% TE sequences, with 233 TEs/Mb (Table 1). Overall, there is approximately 6-fold enrichment in amount of DNA and a 14-fold increase in TE density in pericentromeric regions relative to non-pericentromeric regions. It must be noted, however, that average values of TE content for pericentromeric regions are more variable than for non-pericentromeric regions, because of heterogeneity both within a given pericentromeric region (Figure 1, see below) and among pericentromeric regions on different chromosome arms. For example, the pericentromeric region of chromosome arm 3R had a much lower TE density than other chromosome arms, perhaps relating to the lack of β-heterochromatic sequences in polytene chromosomes at the base of this chromosome arm [27,28]. TE abundance in the pericentromeric region of the X chromosome is likely to be underestimated because of an unsized and unsequenced physical gap in cytological division 20 [9,12], which is embedded in a region of extremely high TE density. Because of these effects and the inclusion of some low TE content regions on 3L and 3R that arise from our use of the high-reduced recombination rate boundary (see above), estimates of TE abundance in pericentromeric regions should be treated as approximate. The rank order of abundance for the major classes of TEs is the same in the pericentromeric regions as in non-pericentromeric regions (% TE sequence: LTR > non-LTR > TIR > *INE-1*; number of TEs/Mb: *INE-1 *> LTR > non-LTR > TIR). Four-fold fewer pericentromeric TEs were full-length (5.1%) relative to non-pericentromeric regions, with 3-fold greater numbers involved in nests (19.5%) (see Table 1). Virtually all TE families (118/121, 97.5%) present in the genome sequence have copies in pericentromeric regions.

#### Chromosome 4

Like pericentromeric regions, the fourth chromosome has a much higher TE abundance than is typical of the genome as a whole: although the fourth chromosome is only 1% of the genome sequence, approximately 10% of TEs annotated are found on chromosome 4. Overall, there is approximately 7-fold enrichment in amount of DNA and a 25-fold increase in TE density on the fourth chromosome relative to regions of normal TE abundance. Important differences in TE abundance between pericentromeric regions and the fourth chromosome were also observed [2,7] (Table 1). Relative to pericentromeric regions, the fourth chromosome has a higher number of TEs per unit of physical distance (422 TEs/MB), but a similar proportion of genome sequence annotated as TE (22.6%). As noted previously [2,7], the rank order abundance of the major TE classes on chromosome 4 differs from the rest of the genome, with TIR elements as the most abundant class of TE (% TE sequence: TIR ~ *INE-1 *> LTR > non-LTR; number of TEs/Mb: *INE-1 *> TIR > non-LTR > LTR). To test the robustness of this pattern, we removed the most numerous family from each of the major TE classes on the fourth chromosome: LTR, *297 *(*n *= 3); non-LTR, *Cr1a *(*n *= 17); TIR, *1360 *(*n *= 62). In the absence of these three highly abundant families, the rank order percent TE sequence (*INE-1 *> LTR > non-LTR > TIR) and number of TEs/Mb (*INE-1 *> TIR ~ non-LTR > LTR) change for the fourth chromosome. This result indicates that patterns of abundance by class on the fourth chromosome are heavily influenced by a few highly abundant families, suggesting that *Cr1a *in addition to *INE-1 *and *1360 *may play an important role in defining the unusual features of this chromosome [18,29]. Fewer TEs on the fourth chromosome are full-length (2.77%) relative to pericentromeric regions, and a lower proportion of TEs are involved in nests (12.6%). Less than half of all TE families (55/121, 45.5%) present in the genome sequence have copies on the fourth chromosome.

Clear differences were also observed in the distribution of TFRs in these three genomic compartments. Consistent with TE densities, non-pericentromeric regions have on average the largest uninterrupted regions of unique sequence (mean 60,320 bp; median 29,280 bp; *n *= 1,663), relative to pericentromeric regions (mean 4,147 bp; median 726 bp; *n *= 2,541) and the fourth chromosome (mean 2,067 bp; median 1,150 bp; *n *= 480). Nevertheless, separate analyses of TFR distributions within each compartment revealed non-random distribution of TEs based on mean TFR lengths in non-pericentromeric regions (adjusted Kolmogorov-Smirnov test, D = 0.1627, p < 0.001), pericentromeric regions (adjusted Kolmogorov-Smirnov test, D = 0.3501, p < 0.001) and chromosome 4 (adjusted Kolmogorov-Smirnov test, D = 0.1541, p < 0.001). We note that finding of non-random distribution of TEs in non-pericentromeric regions in the genome sequence differs from previous conclusions based on cytological estimates [30]. Our results indicate that the non-random distribution of TEs across the entire genome is not explained solely by overall differences in TE abundance between genomic compartments and suggest that the mechanisms that determine the location of TE insertions, such as gene density and ectopic recombination [7,15,31], may be decoupled from overall TE abundance.

### Localized regions of extremely high TE density

With this improved calibration of the background TE abundance that is typical of the major chromosome arms, we sought to identify specific regions of the genome with an extremely high local TE density (we abbreviate such high-density regions as HDRs). We omitted *INE-1 *from this analysis to prevent this very abundant family from dominating the overall genomic trends. Additionally, since it has been postulated that *INE-1 *underwent a burst of transposition prior to speciation and has subsequently become immobilized [16,32], *INE-1 *elements are predicted to be fixed (barring subsequent deletion). As such, their distribution in the sequenced strain should represent a more stable baseline of ancestral TE content to compare with other more recently active TE families. We identified 24 HDRs containing 10 or more (non-*INE-1*) TEs in a 50 Kb window, a cut-off of roughly 20-fold higher density of TEs than the majority of the genome (Figure 1, Table 2). Two HDRs have been previously reported: HDR8 at cytological division 38 [33] and HDR3 at cytological division 20A, which is likely to be fixed in *D. melanogaster *[34].

As expected, nearly all HDRs are located in pericentromeric regions or on chromosome 4, consistent with the general observation that heterochromatic and/or low-recombination rate regions of the genome sequence have high TE densities (see above) [2,7,15]. Three HDRs (1, 16, 17) on the major chromosome arms are located in regions not defined as pericentromeric; however, HDR1 on the X-chromosome is found very close to the boundary demarcating these regions and could probably be classified as pericentromeric. HDRs total 4.27 Mb of sequence and, therefore, comprise only 3.6% of the genome, but contain one-third (1,822/5,390; 33.8%) of annotated TEs. Interestingly, one of the most extreme regions of localized TE density in the *D. melanogaster *genome sequence (HDR4) contains the insertion site for a *P*-element induced allele (*flam*^*py*+(*P*)^) of the as-yet-uncharacterized gene *flamenco *[35], one of the few genetic loci shown to regulate the activity of transposable elements in *Drosophila *[36]. HDR4 (which includes the physical gap in cytological division 20) occupies over 230 Kb of DNA and contains at least 104 TEs and 6 genes, including *DIP1*, which has been excluded as being the gene that is causal for the *flamenco *mutation [35]. We note that the *COM *locus also in 20A2-3, which is known to regulate the *ZAM *and *Idefix *families of LTR elements, is genetically separable from *flamenco *[37] and, therefore, unlikely to correspond to the same region.

**Figure 2:**
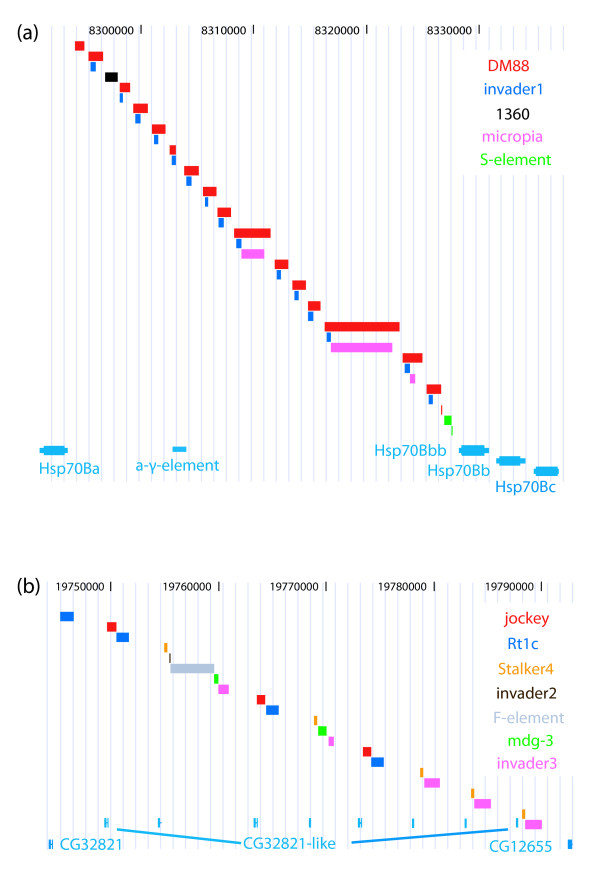
Example regions of extreme TE density. **(a) **Structure of HDR16 in the *Hsp70B *region showing tandem arrays of an *invader1→DM88 *nest interrupted by *1360 *and *micropia *insertions and flanked by *S-element *insertions. Duplicate *Hsp70 *genes are shown at the bottom of the panel along with the non-coding RNA *αγ-element*. **(b) **Structure of HDR1 showing tandem arrays of clustered *jockey*+*Rt1c *and *Stalker4*+*invader3 *elements interrupted by *invader2*, *F-element *and *mdg3 *insertions. This region also generates eight *CG32821*-like gene duplicates. Note that colors for TE families differ in (a,b).

Two exceptional HDRs are found on chromosome arm 3R. HDR16 contains a set of duplicated, nested TEs in the intergenic region between *Hsp70Ba *and *Hsp70Bb *in division 87C (Figure 2a). This region contains the *αβ *repeat [38], which our results indicate corresponds to a duplicated nest of *Dm88 *and *invader1 *sequences (see also [34,39]. The fact that the *αβ *repeat is composed of TE sequences, as predicted by Hackett and Lis [40], explains the observation that components of the *αβ *repeat are dispersed in multiple heterochromatic locations [40] and share homology with 'clustered, scrambled' arrangements of middle repetitive DNA located elsewhere in the genome [41]. This region also contains the non-coding RNA gene known as the *αγ*-*element*, which is transcribed in response to heat shock [38,42] and is a chimeric transcript composed of *Dm88 *and *invader1 *sequences emanating from a fragment of the *Hsp70 *promoter [43]. It is likely that the unusually high abundance of TE insertions in this region has arisen in part because of the unusual chromatin architecture of heat-shock promoters [44,45]. The peculiarity of this region is underscored by the fact that *αβ *repeat has evolved since the divergence of *D. melanogaster *from its sister species *D. simulans *[42,46], but yet appears to be fixed in *D. melanogaster *[47].

The second exceptional HDR (17) on chromosome arm 3R corresponds to a tandemly duplicated array of *invader4 *elements embedded within the sub-telomeric mini-satellites called telomere-associated sequences ('TAS'). We also found that TAS repeats from chromosome arm 2R [48] and the original TAS repeat derived from the *Dp1187 *X-minichromosome [49] also contain *invader4 *sequences (results not shown), although no homology to *invader4 *(or any other TE) is observed in the TAS repeat derived from chromosome arms 2L or 3L [48,50], suggesting that TE sequences are not functionally constitutive components of TAS repeats. The presence of mobile TE sequences in TAS repeats may explain non-telomeric hybridization signal to TAS probes in the chromocenter and basal euchromatic locations [49]. No HDRs are observed at the ends of other chromosome arms, despite the fact that, in *Drosophila*, the retrotransposons *Het-A*, *TART *and *TAHRE *function as telomeric repeats to ensure proper integrity of the chromosome ends [51-53]. In the Release 4 sequence, only the X chromosome and fourth chromosome [9] terminate with small clusters of telomeric TE sequences.

### Mechanisms that generate localized regions of high TE density

Surprisingly, the improved resolution provided by our new annotation showed that TE density is not uniformly high in pericentromeric regions, nor is TE density simply an increasing function of proximity to centromeric regions (Figure 1, inset panels). This is especially true for chromosome arms X, 2L and 2R, where pericentromeric HDRs are interspersed with regions of normal TE density, creating a ragged, punctate increase in TE abundance in the direction of the centromere. Chromosome 4 also exhibits discrete regions of different TE density (Table 2), despite a higher overall level of TE abundance. Some HDRs (for example, 1, 8, 13, 16) clearly occur in regions of low *INE-1 *density, which suggests a recent origin for the high TE density in these regions, assuming that *INE-1 *represents the ancestral TE distribution at the time of its major burst activity prior to the split of *D. melanogaster *from its sister species *D. simulans *[16,32]. Other HDRs (9, 10, 15 and those on the fourth chromosome) co-occur with regions of high *INE-1 *density, suggesting these regions of the genome have permitted a high density of TEs, at least as far back as the ancestor of the *D. melanogaster *species subgroup [16,32]. This also is likely to hold true for HDRs 11, 12 and 14 at the bases of chromosome arms 2L, 2R and 3L, where non-*INE-1 *TEs occupy virtually all of the sequence, creating an apparent negative association with *INE-1 *density.

**Figure 3:**
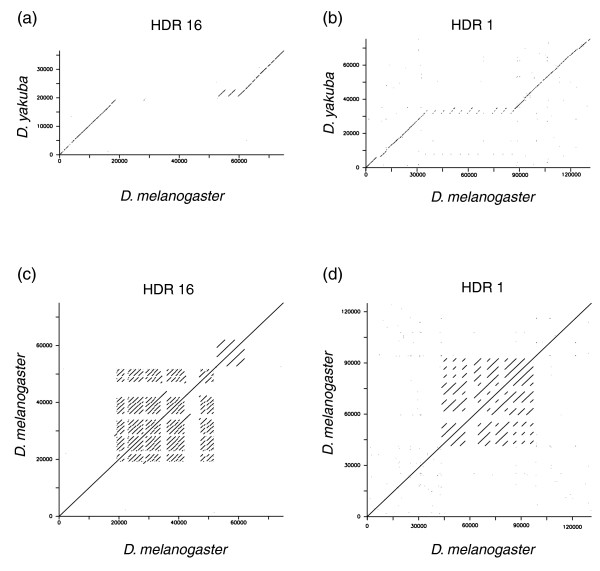
Comparative sequence analysis of two regions of extreme TE density. **(a,b) **Pairwise comparison of *D. melanogaster *HDRs with the orthologous segments from the *D. yakuba *genome. **(c,d) **Self-comparison of *D. melanogaster *HDRs. Note that the flanking sequences between species are collinear (a,b) and the presence of complex duplicated sequences (c,d).

What evolutionary mechanisms cause such a localized pattern of extreme TE density? Clearly, transposition is the ultimate source of all TE insertions in the genome, and accordingly HDRs typically contain a mix of different TE families and nested elements (Table 2), both hallmarks of recurrent transposition. However, it is possible that other mechanisms of genome evolution - such as inversion or duplication - might have contributed to the origin of HDRs. To investigate whether this punctate pattern of HDRs arose from chromosomal inversions that bring TE-rich, heterochromatic DNA into euchromatic regions, we extracted orthologous regions from the *D. yakuba *genome sequence and assayed whether the unique sequences flanking HDRs are collinear in the two species. We found that unique sequences flanking HDRs were collinear for 15 of the 16 HDRs (93.8%) that are internal to the ends of the chromosome arms, for which both flanking sequences can unambiguously be identified (Table 2, Figure 3a,b). Intriguingly, HDR 13 does occur in the same region as an inversion breakpoint between *D. melanogaster *and *D. yakuba*, but outgroup analyses place this inversion event on the *D. yakuba *lineage, not the *D. melanogaster *lineage (JM Ranz, D Maurin, YS Chan, LW Hillier, J Roote, M Ashburner and CM Bergman, personal communication). Thus, we found no evidence indicating that inversions carrying TE-rich DNA from heterochromatic regions generate HDRs, but remarkably we did find evidence that a region of the *D. melanogaster *genome that permits a high TE density can tolerate inversion breakpoints in other *Drosophila *lineages. It is important to note, however, that the majority of HDRs do not correspond to inversion breakpoint regions and *vice versa*.

We did, however, find a relatively high incidence of duplicated sequences in HDRs, suggesting that tandem or segmental duplication plays an important role in the genesis of TE-rich regions of the genome: 13 of 23 HDRs show evidence of duplication (Table 2, Figures 2 and 3c,d). Duplications in HDRs can contain multiple TEs from different families, often nested, sometimes with different copies of the duplicated region containing additional TE insertions (Figure 2). Duplications in HDRs also amplified cellular genes as well as TE sequences: for example, eight partial and complete duplicates of the gene *CG32381 *are present in HDR1 (Figure 2b). HDRs may also include retrotransposed gene duplicates, such as the *Mgst1*-like *CG12628 *[54], which is found in a nest of TEs in HDR11. The series of events leading to tandem duplication of TEs in HDRs is often highly complex, with repeat structures present at different scales (Figure 3c,d). Duplication of TE sequences could also be observed in other regions of the genome with lower TE density, such as duplication of *Rt1c *elements interspersed between the *SDIC *gene duplicates [55,56]. A more thorough analysis of the interplay between TEs and segmental duplications will be the subject of a separate study (A-S Fiston, D Anxolabehere and H Quesneville, personal communication).

### A graph-based approach to analyze patterns of TE nesting

Regions of extremely high TE density typically contain a high proportion of TEs inserted into other TEs, and our new annotation allowed us to examine patterns of TE nesting in greater detail than has previously been possible. Few methods exist to analyze TE nesting, partly because of limitations in accurately joining fragments of a TE insertion that become separated in the genome by a subsequent nested TE insertion, and partly because analysis of TE nesting is complicated by the redundancies inherent in complex nesting relationships. For example, if one TE (A) is nested within a second (B) that is in turn nested within a third (C), simply analyzing overlapping ranges of TEs in the genome will erroneously yield three nesting events (A→B, A→C, and B→C), when only two occurred historically (A→B and B→C). We found that complex nesting relationships could best be analyzed by identifying 'primary' nesting relationships (A→B and B→C in the example above) and assembly of these simple binary events into more complex nesting relationships by applying concepts from network analysis to describe and quantify patterns of TE nesting. In this formulation of the problem, TE nesting relationships are represented as a graph having TEs as nodes and transposition events as directed edges. The directed nature of this graph implies both the spatial relationships of nested TEs in the genome as well as temporal relationships implied in TE nesting resulting from the fact that the outer TE in a nest must have existed in the genome prior to the insertion of the inner TE [57]. This 'nesting graph' is amenable to standard computation and can be recast in several forms, since each annotated TE node can be analyzed at the individual, family or class level. (We chose not to analyze the degree of distribution of nesting graphs for 'small-world' properties because of biases resulting from duplicated nests, and because the subgraphs in the sequenced portion of the genome may not reflect properties of the entire nesting graph [58].)

**Figure 4:**
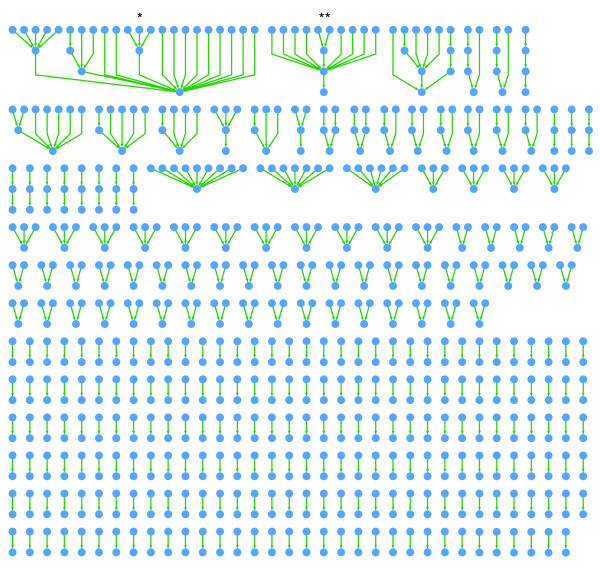
Global nesting graph at the level of individual TEs. Nesting relationships among TEs are depicted as a directed, acyclic graph. Nodes (blue circles) represent individual TEs and directed edges (green arrows) represent transposition events that create primary nesting relationships, with complex nesting events represented as connected components of the graph. The majority of nests in the genome are characterized by one or more primary nesting relationships, while some larger nests are composed of secondary or tertiary nesting relationships. The largest nest (*) currently annotated in the genome is found on chromosome 2R at coordinates 1,763,561-1,829,561. The second largest nest (**) currently annotated in the genome has been described in detail previously by Maside *et al*. [34] and is found on chromosome X at coordinates 21,366,773-21,333,853.

At the individual TE level, nesting relationships form a sparse, acyclic graph of 785 nodes and 491 edges that provides a detailed overview of the global pattern of TE nesting in the *D. melanogaster *genome (Figure 4). These 785 TEs (14.6% of all 5,390 TEs annotated) are found in 294 distinct nests, which can be calculated from the number of sink TEs (nodes in the graph that have an out-degree of zero). These 294 nests are formed by 448 source TEs (nodes in the graph with an in-degree of zero), and 43 TEs that act as internal nodes in the graph (with both non-zero in-degree and out-degree). The vast majority of TE nests in *D. melanogaster *(263/294, 89.4%) are composed of simple 'primary' nests with a maximal path length of one, consisting mainly of one (203/263, 77.2%) or sometimes greater than one (60/263, 22.8%) inner TE nesting into a single outer TE. Of the 31 nests with more complex nesting relationships, 25 have a maximal path length of two ('secondary' nests), and only 6 have a maximal path length of three ('tertiary' nests) (Figure 4). Relative to the proportion of the genome in each compartment, nests are highly enriched in pericentromeric regions (215/294, 73.1%), as well as on the fourth chromosome (27/294, 9.2%), but rare in non-pericentromeric regions (52/294, 17.7%).

**Figure 5:**
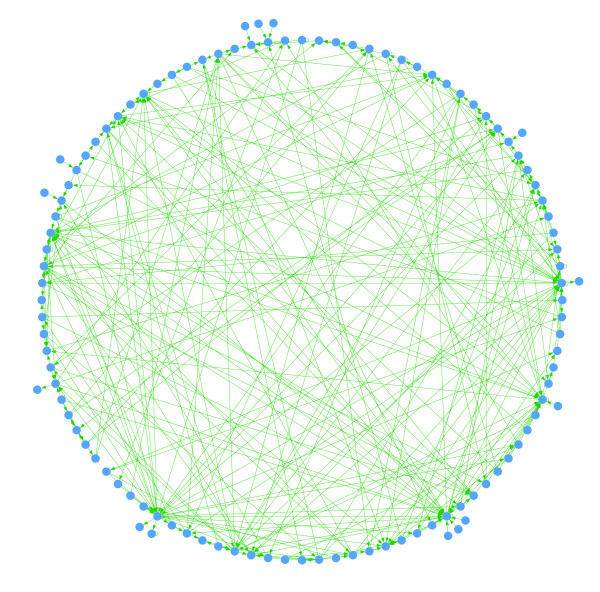
Global nesting graph at the level of TE families. Nodes (blue circles) represent TE families and directed edges (green arrows) represent observed instances of primary nesting relationships. Redundant edges that arise from the different instances in the genome of the same primary nesting event are not shown. Essentially all families of TEs form a single connected component. Note that cycles within and between families at the family level are formed from nests of individuals from different genomic locations.

The nesting graph at the individual level provides details about the structures of all TE nests in the genome, but since individual TEs are members of distinct families nesting relationships can also be analyzed at the family level by relabeling nodes with family identifiers and collapsing redundant edges. Recasting the same set of TEs as a nesting graph at the family level provides novel means to study the physical proximity and historical co-existence of all TE families at a global level. Nesting relationships at the family level form a highly connected cyclic graph of 110 nodes and 334 edges (Figure 5), involving the vast majority (90.1%) of the 121 TE families represented in the Release 4 genome annotation. This result implies that nested TEs provide paths of sequences that connect virtually all families, and that a large diversity of novel chimeric sequences between different families exists in the junction regions between TEs in nests. Most TE families (80/110, 72%) are internal nodes in the graph acting as both inner and outer components of nests, with only 22 source families and 8 sink families. The majority of families (97/110, 88%) also form nested relationships with more than one other family. Fifteen families have members that transpose into another member of same family, forming self-loops (or cycles) in the graph. Self-nests require a genomic copy to be present into which another family member can insert, and are consistent with multiple bursts of transposition for a given family or a burst that extends over multiple host generations.

**Figure 6:**
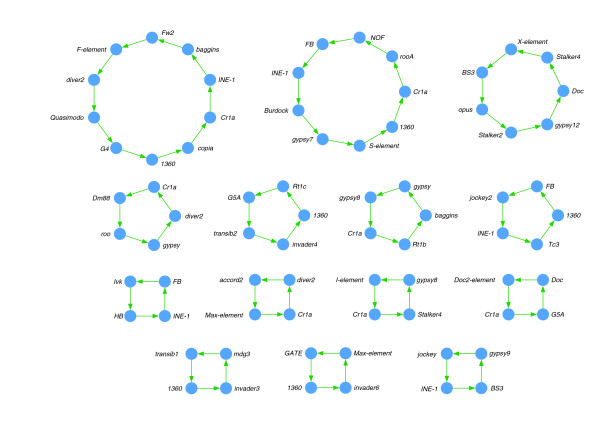
Directed cycles in the family-level TE nesting graph. Shown are the set of edge-disjoint directed cycles of path length greater than three. Nodes (blue circles) represent TE families and directed edges (green arrows) represent observed instances of primary nesting relationships. Note that many thousands of distinct directed cycles that share edges in common can be enumerated in the family-level nesting graph in addition to those shown here.

Directed cycles other than those from self-nests were also observed in the family-level nesting graph, clearly indicating either continuous or discrete periods of overlapping transpositional activity for different TE families in the lineage leading to *D. melanogaster*. Exhaustive enumeration detected 12 distinct cycles of length two (A→B→A), and 43 distinct cycles of length three (A→B→C→A), in the family-level nesting graph, with tens of thousands of distinct cycles of length less than ten. The complexity of the family-level nesting graph is such that it is not feasible to enumerate all cycles in reasonable time; however, a set of independent cycles that do not use the same edge can be extracted efficiently. Figure 6 shows the set of edge-disjoint cycles of length greater than three in the family-level nesting graph, and provides examples of the complex periods of contemporaneous TE activity that must be invoked to explain the global pattern of nesting at the family level. These procedures detect many novel examples of nesting among families, in addition to classical examples such as *NOF→FB *nesting [59,60].

The complexity of nesting among TEs observed at the individual and family levels simplifies at the class level (Table 3). The nesting graph at the class level is complete save for events involving the rare Foldback (*FB*) class of TEs, with instances of all possible types of nesting between LTR, non-LTR, TIR and *INE-1 *elements observed in the genome. The most frequent type of nesting event at the class level is LTR→LTR (151/491, 30.7%) and LTR elements form both the most frequent inner (233/491, 47.4%) and outer (207/491, 42.1%) components of nests, extending the finding based on Release 3 that LTR elements are most often involved in nests or clusters [2]. The rank order of abundance for both inner and outer members of nests is LTR > non-LTR > TIR > *INE-1 *> *FB*, which follows the trend for amount of TE sequence in the genome by class rather than number of TEs, indicating that target size influences class level nesting patterns (Table 1). The observed number of nests for pairwise combinations of classes departs significantly from the random expectation based on the marginal counts of inner and outer nests for each class (χ^2 ^= 144.9, 16 degrees of freedom (df), p < 10^-16^). Non-random patterns of nesting are observed just for the three major classes of TEs (χ^2 ^= 81.8, 4 df, p < 10^-16^), suggesting that neither the low *FB *counts nor undue influence by *INE-1 *causes this pattern. The non-random pattern of nesting appears in large part to be the result of preferences for TEs to nest in other TEs of the same class, which may represent some sort of a 'homing effect' mediated through protein complexes shared by the TEs belonging to the same class. Alternatively, the non-random pattern of nesting among TE classes may also result from complex historical factors, including the total amount of pre-existing TE sequence present in the genome as targets for insertion, and/or a non-random order of transpositional events among families and classes of TEs.

## Discussion

### Organization of TEs in β-heterochromatic regions

The nature of the transition zone between euchromatin and heterochromatin in *D. melanogaster *has been the subject of much controversy, in part because heterochromatic regions (as defined in mitotic chromosomes) can be further subdivided into α-heterochromatin and β-heterochromatin [61]. β-heterochromatic regions are cytologically visible in polytene chromosomes, although their banding pattern is 'diffuse' or 'mesh-like,' suggesting under-replication relative to the finely banded euchromatic regions (reviewed in [28]). Under-replicated regions are observed elsewhere in polytene chromosomes and co-localize with regions referred to as 'weak points' or 'intercalary heterochromatin' that form ectopic contacts and are subject to chromosome breakage [62,63]. The amount and degree of polytenization in β-heterochromatic regions is subject to both environmental and genetic factors [64], as most conclusively shown by the appearance of several large banded regions in the chromocenter of salivary gland chromosomes of the *Su*(*UR*) mutant [65]. Charlesworth *et al*. estimate that 10% of the *D. melanogaster *genome is composed of β-heterochromatin [24] and large amounts of β-heterochromatic DNA are found in pericentromeric regions of most (but not all) chromosome arms [27,28], a fraction of which is captured in the Release 4 genome sequence (Figure 1).

Analysis of the first draft of the *D. melanogaster *genome sequence offered the first glimpse of the contiguous molecular organization of β-heterochromatin, and suggested that "there is no clear boundary between heterochromatin and euchromatin" but rather that the transition is characterized by "a gradual increase in the density of transposable elements and other repeats" [66]. The view that the β-heterochromatic regions exhibit a gradual increase in TE density has been subsequently reiterated [25,67], although our results call this view into question for three of the five major chromosome arms. Far from a gradual transition, our high-resolution TE annotation provides evidence for discretely localized regions of extremely high TE density at the base of chromosome arms X, 2L and 2R overlain on a background of increased TE abundance, such that the increase in TE content is not monotonic in the direction of the centromere. This result represents the inverse of, and provides an explanation for, previous observations that the distribution of genes on these chromosome arms alternates between low and high density in the centromere proximal direction [66,67]. We note that the alternating pattern of high and low TE (versus unique) sequences reported here in β-heterochromatic regions differs from the 'islands' of complex (TE) sequences surrounded by 'seas' of satellite DNA observed deeper in α-heterochromatic regions [68].

**Figure 7:**
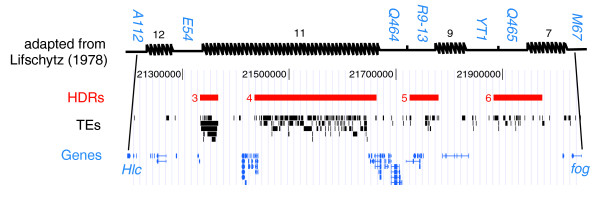
HDRs are hotspots for X-ray induced deletion. Alignment of the genetic map adapted from Figure 1 of Lifschytz [69] and the Release 4 genome annotation in the interval from *Hlc *(= *A112*) to *fog *(= *M67*) shows a one-to-one correspondence between HDRs 3, 4, 5 and 6 with X-ray hotspot intervals 12, 11, 9 and 7, respectively. Additional HDRs and X-ray hotspots discussed in the text are omitted for clarity.

How does the ragged, punctate pattern of TE density affect the interpretation of the transition zone between euchromatin and heterochromatin? If TE density is directly responsible for heterochromatin formation, then such discrete regions of extreme TE density may argue against a gradual transition between euchromatin and heterochromatin, and would support the model of Lifschytz [69], who suggested several distinct transitions between euchromatin and heterochromatin in cytological divisions 19-20 of the X chromosome. However, as noted by Yamamoto *et al*. [70], the interpretation of multiple, discrete transitions between euchromatin and heterochromatin by Lifschytz [69] was based indirectly on the distribution of X-ray induced deletions, rather than direct cytological evidence for heterochromatic properties. We integrated our annotation of HDRs in the Release 4 genome sequence with the genetic map of Lifschytz [69] and found a striking correspondence between our HDRs and 'hotspots' for X-ray induced deletions in his analysis. Based on the few complementation groups that can be mapped to the genome *(A112 *= *Hlc*, *M67 *= *fog*, and *X-3 *= *stn*), we hypothesize that our HDRs 2, 3, 4, 5, 6, and 7 correspond to hotspot intervals 18, 12, 11, 9, 7, and 6, respectively, in [69] (Figure 7). The major hot-spot for X-ray induced breakage in this region (interval 11 in 20A) most likely corresponds to HDR4, which we find to be the region of highest TE density in the genome sequence. Together, these results suggest that the Lifschytz [69] data may simply reflect preferential breakpoint use in TE-rich regions devoid of genic function, rather than multiple distinct transitions between euchromatin and heterochromatin. These conclusions support those of Ashburner *et al*. [71], who showed that the distribution of rearrangement breakpoints in the *Adh *region correlates with the amount of DNA in an interval rather than with any property of the sequence itself.

Further evidence that discrete regions of extreme TE density outside of β-heterochromatic regions may have unusual cytological properties can be found on chromosome 2. Discrete HDRs can be observed in the vicinity of the *Histone *cluster in 39E (HDRs 9+10) and just distal to the major tRNA cluster at 42A (HDR13). Both of these regions are known to be 'weak points' in polytene chromosomes, which form breaks and ectopic contacts with other weak points in the genome that are alleviated by the *Su*(*UR*) mutation, suggesting that these regions are under-replicated in polytene chromosomes [65]. These observations, together with the generally poor banding patterns in high TE density pericentromeric regions and on the fourth chromosome, suggest that high TE density may directly interfere with the process of polytenization, either through stalling replication forks [72] or through DNA elimination [73]. Thus, high TE densities may not be directly responsible for heterochromatin formation *per se*, but may simply inhibit the ability to detect *bona fide *euchromatic regions that are TE dense, at least in salivary gland polytene chromosomes. The formation of large blocks of TE-rich, banded material deep in heterochromatic regions in under-replication suppressing strains like *Su*(*UR*) supports this view [65,74]. Moreover, if regions of high TE density affect polytenization, ectopic contact among 'weak points' may occur *via *homology between sequences of the same TE family. Additionally, the inherent mobility of TEs provides a mechanism to explain differences in the presence or absence of β-heterochromatin on homologous chromosome arms among *Drosophila *species [28].

### Origin of 'clustered scrambled repeats'

Although the predominant organization of middle repetitive DNA such as TE sequences in *D. melanogaster *is characterized by individual repeats found within long regions of single copy DNA (the 'long period interspersion' pattern) [21], direct evidence has long existed for an alternative organization characterized by 'clustered scrambled repeats' [21,41]. Wensink *et al*. [41] estimated that the genome of *D. melanogaster *contained over 1,000 such clustered scrambled repeats and predicted that these regions were created by recurrent mobile element insertion. The HDRs and TE nests detected in the present study likely correspond to a subset of the clustered, scrambled repeats detected by Wensink *et al*. [41], with the remainder yet to be discovered in currently unfinished or unsequenced heterochromatic regions. Clustered, scrambled TE nests are generally thought to arise through the serial transposition of individual elements into previously inserted TEs, as shown by the analysis of nested TEs in maize, which demonstrated that the ages of inner TEs are younger than the outer TEs into which they insert [57]. Such serial transposition is ultimately responsible for the origin of nested TEs, though once formed, nests may be subsequently copied and amplified. Therefore, it is possible other mechanism may play an important role in the genome-wide pattern of clustered scramble repeats, such as the transposition or duplication of previously nested elements.

**Figure 8:**
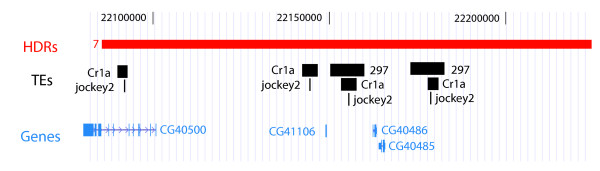
Examples of potentially transposed TE nests. Four copies of related *jockey2→Cr1a *nests in HDR7 at the base of the X chromosome, with the two proximal copies nested within *297*-elements. We note that a large number of additional TEs in this region are omitted for clarity. Simple tandem duplication of *jockey2→Cr1a *nests cannot explain nesting in the *297*-element, and duplication of a *jockey2→Cr1a→297 *nest would require two subsequent complete losses of 297 sequences from the distal copies. An equally or more parsimonious explanation involves transposition of a *jockey2→Cr1a *nest into a *297*-element and subsequent duplication.

Evidence for duplication of clustered or nested TEs is clearly observed in our data (Figures 2 and 3), as was predicted by Charlesworth *et al*. [24], who argued for duplication of TEs on the basis of high variance in heterochromatic TE copy number among strains of *Drosophila*. Duplication of clustered or nested TEs has also been reported previously in heterochromatic regions of the *Drosophila *genome not studied here [39,68,75], as well as in the barley [76,77] and *Arabidopsis *[78] genomes. Although the phenomenon of duplicated clusters/nests in TE dense regions appears to be a recurrent pattern, the exact mechanism(s) that create duplicated clusters/nests is (are) less clear. In some cases, tandem duplication is sufficient to explain the pattern of identical or related TE clusters/nests in the same local region (for example, the *Hsp70 *region). However, some examples of duplicated clusters/nests do not seem to fit with a model of simple tandem duplication. One such example is found in four identical instances of a *jockey2→Cr1a *nest at the base of the X chromosome in HDR7 (Figure 8). The two proximal copies of this repeated nest are themselves nested within *297 *elements (FBti0062438→FBti0062418→FBti0062352;FBti0062439→ FBti0062435→FBti0062353) and are separated by unique sequence containing the genes *CG40485 *and *CG40486*. The two distal copies of this nest are not inserted into *297 *or any other TE (FBti0062436→FBti0062415; FBti0062437→FBti0062417) and are separated from each other by approximately 45 Kb of other TE and unique sequences, with the most distal copy found in the intron of the gene *CG40500*. No evidence of tandem duplication can be observed in comparisons of the *D. melanogaster *region with itself or the orthologous *D. yakuba *region (results not shown), nor is there any relic of *297 *sequence surrounding the two distal copies, as would be expected if they arose by simple tandem duplication.

Such observations are difficult to explain without proposing that the duplicated copies of this *jockey2→Cr1a *nest arose by transposition of a pre-existing nested element, as was proposed to occur by Wensink *et al*. [41]. Other potential examples of transposition of clustered, scrambled repeats can also be observed in our data, such as a *jockey*-*Rt1c *cluster present in both HDR1 and HDR2, which are separated by over a megabase of DNA, and a *BS3*-*X-element *cluster present near *su*(*f*) and that is also found in HDR7 [39]. Though it may seem unlikely, the transposition of nested TEs is indeed plausible since DNA-based elements can transpose when additional sequences are inserted between TIRs [79], and retroelements may reverse transcribe mRNAs arising from nested or rearranged TEs, a mechanism that has been invoked previously for the formation of new TE families [80]. Moreover, the raw material for retrotransposition of nested elements is available in the fly transcriptome, as reflected in the chimeric transcripts that arise from two or more TEs found in *D. melanogaster *EST/cDNA libraries (results not shown).

### Do β-heterochromatic regions permit the evolution of co-suppression networks?

A growing body of evidence implicates RNA silencing mechanisms in regulating the activity of TE expression and transposition in *Drosophila*. Expression of TE-derived transcripts is elevated in mutations for genes involved in RNA silencing, including *spn-E*, *aubergine *and *piwi *[81-84]. The capacity of telomeric *P-element *insertions to induce the repressive *P*-cytotype is also impaired in *aubergine *mutants [85]. All major classes of TE in *Drosophila *produce small repeat associated RNAs (rasiRNAs) [86] that may be used to silence TE expression using a *dicer*-independent RNA silencing pathway [84]. Moreover, the Argonaute family member *piwi *regulates expression from *copia *and *gypsy *reporter transgenes [82,83] and rates of *mdg1 *transposition are elevated in a *piwi *mutant background [82]. Similarly, resistance to invasion by the *I-element *can be provided by strains carrying a transgene containing *I-element *sequences in a dose-, length- and transcription-dependent manner [87]. Heterologous reporter genes carrying transcribed *gypsy *sequences are also sensitive to regulation by *flamenco *[83], suggesting the possibility of an RNA dependent mechanism of action for this locus, which is known to regulate rates of *gypsy *transposition.

Regulation of TE transposition may rely on endogenous TE sequences present in the genome as well as the RNA silencing machinery. Jensen *et al*. [88] proposed an indirect model of co-suppression through 'relay' sequences derived from defective *I-elements *located in pericentromeric regions. Likewise, mapping of factors controlling rates of *gypsy*, *ZAM *and *Idefix *transposition to a β-heterochromatic location at the base of the X chromosome has led Desset *et al*. [89] to propose that transcription from remnants of TEs in 20A may provide the critical substrate for co-suppression of these transposable element families. Our work demonstrates that the *Drosophila *genome contains ample material for co-suppression within virtually all TE families, given the fact that transcription is known to occur in β-heterochromatin regions [90].

In addition to the possibility of co-suppression among different copies of the same TE family, our analysis of nesting relationships among different TE families suggests the possibility of an extensive network of co-suppression among essentially all families in the genome (Figure 5). We propose that expression of chimeric sequences from TE nests may simultaneously co-suppress multiple TE families by acting as relay sequences that co-suppress transcripts from other nests or individual elements located in the euchromatic arms. Evidence for such a 'co-suppression network' is found in the *COM *locus, which appears to control the activity of more than one TE family simultaneously. Even in the absence of direct co-suppression on a family, once a member of a newly invading TE family transposes into the nesting network, the entire family could become regulated by co-suppression mechanisms. This model proposes that the accumulation of clustered, nested TEs in β-heterochromatic regions may incidentally provide a trap for the regulation of TEs across the genome, and solves the need for the host to evolve separate genic changes to regulate the transposition of each new family that invades the genome. Such a co-suppression network could act as a global TE surveillance mechanism, with the highly nested structure of TEs in β-heterochromatic regions intrinsically providing a systems-level 'adaptive immunity' from invasion by horizontal transfer. Moreover, since nesting can bring several TE promoters in close proximity to each other and thereby increase the probability of transcription, TE nesting may facilitate a more effective co-suppression network than would be possible through the cumulative effects among isolated TEs within single families. Finally, since as a TE family increases in number the chance it participates in the co-suppression network is likely to increase, pervasive nesting may also generate a pressure on TE families to diversify (as has previously been proposed for the mechanism of ectopic recombination [91]), potentially providing an explanation for the large diversity of TE families in the *Drosophila *genome.

## Conclusion

By accounting for the non-random distribution of TEs across the genome, we provide an accurate estimate of TE abundance for the vast majority of the genome sequence in high-recombination, non-pericentromeric regions. We confirm that regions of extreme TE density are mostly in the pericentromeric and/or low-recombination regions of the genome that are known broadly to have high TE abundance. However, we show that regions of high TE density within pericentromeric regions are often distinctly localized and interrupted by regions of normal TE density in the transition zone from euchromatic to β-heterochromatic regions. Through comparative analysis with *D. yakuba*, we found no evidence that this ragged, punctate pattern of highly localized TE abundance arises *via *inversion of TE-rich sequences from deeper in heterochromatic regions. We did find, however, that duplication of TE sequences plays an important role in the rapid evolution of localized regions of extreme TE abundance. We introduced network analysis techniques to study patterns of TE nesting, providing a comprehensive view of the spatial and temporal interactions among TEs at the individual, family and class levels. We show the existence of a highly connected family-level nesting network, which suggests the possibility of an intrinsic 'co-suppression network' acting to regulate the vast majority of TE families in *D. melanogaster *genome. The results presented here provide a framework for comparison with finished heterochromatic sequences being produced by the *Drosophila *Heterochromatin Genome Project [13].

## Materials and methods

### Dataset of TE annotations

The combined-evidence method used to identify TE sequences has been described previously [10]. Briefly, borrowing concepts from gene annotation, we have developed a TE annotation pipeline that integrates multiple lines of computational evidence to generate 'TE models.' The 6,013 predicted TE models of Quesneville *et al*. [10] were used with the following exceptions. Three TE annotations were removed (FlyBase IDs: FBti0062904, FBti0060950 and FBti0060875) that have subsequently been shown to be redundant entries that resulted from edge effects in overlapping contigs. In addition, all TE models based on non-*D. melanogaster* canonical elements were removed with the exception of those from *D. simulans*, the sister species to *D. melanogaster*, to be conservative in our analyses. These 620 annotations from foreign elements account for over 10% of the TE models but only 82,229 bp (1.2%) of sequence of the sequence annotated as TE in [10].

### Testing a model of random TE distribution

Under the null hypothesis that TEs are distributed uniformly throughout a genomic region, distances between TEs (TE-free regions, abbreviated as TFRs) should follow a negative exponential distribution [20]. In contrast to the analysis of Simons *et al*. [20] who evaluate the number of TFRs above an arbitrary length cutoff, we test the fit of observed TFR lengths to the full negative exponential distribution. The rate parameter for the negative exponential can be estimated in two ways, either as the inverse of the mean of observed TFR lengths, or by dividing the number of TE insertions by the total length of TFRs, as in [20]. In the first case, the observed TFR distribution can be tested directly against the expected distribution computed from the negative exponential distribution. In the second case, since nested elements contribute to the number of TE insertions but not the length of TFRs, the number of inner nested TEs (491) must be discounted from the total number of insertions before computing the average TFR length, or an equivalent number of 0-length TFRs must be added to the observed TFR length distribution. Goodness-of-fit to the negative exponential distribution was calculated using the Kolmogorov-Smirnov one-sample statistic in R [92], which computes the maximal difference between the observed and expected cumulative distributions. We have used adjusted critical values taken from [22] to account for the fact that the rate parameter of the expected distribution was estimated from the data.

### Definition of chromatin and recombination boundaries

Cytological boundaries of the pericentromeric euchromatin/heterochromatin boundary were estimated from the mitotic FISH data in [25], as mapped to Release 4 (Chris D Smith, personal communication). Boundaries between 'high,' reduced,' and 'null' recombination rates in pericentromeric regions [26] were estimated by mapping cytological locations to the Release 4 sequence using the 'cytolocation' search in FlyBase gbrowser [93]. Ranges of cytological divisions were grouped into genome coordinates following Bartolome *et al*. [7]. Boundaries of pericentromeric regions were operationally defined for the major chromosome arms as regions of reduced recombination as the proximal positions of bands 19D3 on chromosome arm X (20,369,021), 38A1 on chromosome arm 2L (19,669,505), and 77E1 on chromosome arm 3L (20,545,022), and the distal positions of bands 42F3 on chromosome arm 2R (2,692,485) and 84B1 on chromosome arm 3R (2,811,816).

### Definition and analysis of regions of high TE density

Sliding window analysis to identify HDRs was done using 50 Kb windows with a 25 Kb offset. The number of TEs per window, rather than percent TE sequence, was used to identify regions of high TE density. Windows having 10 or more TEs/50 Kb (that is, 200 TEs/Mb), a density that is approximately 20-fold the average of non-pericentromeric regions were used to seed HDRs. Neighboring windows were then merged to define the final set of HDRs, allowing intervening windows of 9 or more TEs/50 Kb to account for small fluctuations in TE abundance. Orthologous regions in the *D. yakuba *(droYak1, April 2004) genome sequence of HDRs in *D. melanogaster *± 50 Kb were extracted from the Berkeley pipeline whole-genome alignments [94] and updated to the most recent version of the *D. yakuba *genome sequence (droYak2, November 2005) using BLAT [95]. Dotplot analysis of orthologous regions was performed on both forward and reverse strands or HDRs and their orthologues using the dottup program in the EMBOSS package [96].

### Graphical analysis of TE nesting

Patterns of TE nesting were analyzed using network analysis techniques, with nesting events represented as directed edges between two TE nodes. The Release 4 annotation represents nested TEs as overlapping spans among sets of genome coordinates, where the range of an inner TE in a nest is fully subsumed within the range of an outer TE. For each inner TE in the genome that met these conditions, we identified the 'primary' nesting relationship among the single outer TE immediately present on both sides of the inner TE span, and created a directed edge in the nesting graph labeled inner→outer. The inner and outer labels were individual, family or class identifiers, depending on the biological level of analysis. These primary nesting relationships provide a sufficient and non-redundant description of TE nesting in the genome, and can be used to reconstruct more complex nesting relationships at the individual, family or class levels. Manipulation, analysis and visualization of nesting graphs were conducted using the PERL Graph module version 0.69 [97], the Combinatorica package in Mathematica 5.1 [98] and Cytoscape 2.2 [99]. Enumeration of all directed cycles of a fixed path length was performed using the method described in [100].

## Figures and Tables

**Table 1:** Abundance of *D. melanogaster *TEs annotated in Release 4 genome sequence by genomic region

	Class	Total bp TE	% TE	No. of TEs	No. of TE per Mbp	No. of TE full length	% TE full length	No. of TE nested	% TE nested
Genome	LTR	3,896,903	3.29	1,321	11.16	325	24.60	327	24.75
	Non-LTR	1,502,997	1.27	1,019	8.61	121	11.87	197	19.33
	TIR	559,234	0.47	752	6.35	57	7.58	157	20.88
	*INE-1*	490,996	0.41	2,238	18.91	26	1.16	91	4.07
	*FB*	60,509	0.05	60	0.51	1	1.67	13	21.67
	Total	6,510,639	5.50	5,390	45.54	530	9.83	785	14.56
									
Non-pericentromeric	LTR	2,510,569	2.42	515	4.96	250	48.54	80	15.53
	Non-LTR	646,020	0.62	336	3.24	80	22.92	9	2.68
	TIR	151,997	0.15	214	2.06	25	11.68	12	5.61
	*INE-1*	106,597	0.10	660	6.36	5	0.76	8	1.21
	*FB*	28,125	0.03	23	0.22	1	4.35	3	13.04
	Total	3,443,308	3.32	1,748	16.85	361	20.48	112	6.41
									
Pericentromeric	LTR	1,324,428	9.94	776	58.24	70	9.02	241	31.06
	Non-LTR	802,040	6.02	623	46.75	42	6.58	169	27.13
	TIR	323,226	2.43	436	32.72	29	6.65	115	26.38
	*INE-1*	300,615	2.26	1,234	92.61	17	1.38	71	5.75
	*FB*	27,773	0.21	32	2.40	0	0.00	9	28.13
	Total	2,778,082	20.85	3,101	232.72	158	5.06	605	19.51
									
Chromosome 4	LTR	61,906	4.83	30	23.41	5	16.67	6	20.00
	Non-LTR	54,937	4.29	60	46.82	3	5.00	19	31.67
	TIR	84,011	6.55	102	79.59	3	2.94	30	29.41
	*INE-1*	83,784	6.54	344	268.41	4	1.16	12	3.49
	*FB*	4,611	0.36	5	3.90	0	0.00	1	20.00
	Total	289,249	22.57	541	422.12	15	2.77	68	12.57

Overall abundance was partitioned into pericentromeric and non-pericentromeric regions according to the text. Full-length elements were defined as ± 3% of the canonical element. Both inner and outer components of a TE nest were considered nested.

**Table 2:** Regions with extreme TE density in the *D. melanogaster *Release 4 genome sequence

HDR	Chromosome	Start	End	No. of families	No. of TEs	No. nested	Duplicated TEs	Collinear	Genes
1	X	19,744,508	19,790,060	7	22	0	+	+	2 (8)
2	X	20,958,143	20,988,686	13	18	2	+	+	1
3*	X	21,332,555	21,366,773	13	14	13	-	+	0
4^†^	X	21,434,542	21,663,556	42	104	39	+	+	6
5	X	21,726,082	21,780,371	10	12	4	-	+	5
6	X	21,883,728	21,974,732	16	21	0	-	+	0
7	X	22,085,438	22,224,390	19	38	12	+	Base	9
8	2L	20,100,865	20,210,447	27	61	18	+	+	1
9^‡§^	2L	21,312,749	21,403,782	20	29	6	+	+	7 (3)
10^‡§^	2L	21,527,053	21,725,165	36	55	17	-	+	10 (1)
11	2L	22,064,386	22,407,834	61	157	52	+	Base	19 (1)
12*	2R	387	1,185,590	103	571	156	+	Base	45
13^§^	2R	1,744,145	2,011,104	42	92	46	+	-	2
14	3L	22,910,473	23,771,865	91	411	128	+	Base	17
15	3R	310,015	436,430	22	37	8	-	+	9
16*	3R	8,294,200	8,327,684	5	38	33	+	+	1
17	3R	27,888,358	27,905,053	2	20	12	+	Tip	1
18	4	1	46,860	12	14	4	-	Base	2 (2)
19	4	201,177	269,428	10	16	9	-	+	6
20	4	303,028	348,412	7	10	2	-	+	4
21	4	433,967	496,527	10	20	7	+	+	4
22	4	926,385	997,041	12	18	3	-	+	5
23	4	1,163,173	1,281,586	18	44	13	-	Tip	9

HDRs were defined as having >10 non-*INE-1 *TEs in a 50 Kb window. Numbers of distinct families, numbers of TEs, number of TEs involved in nests, and the presence of duplicated TEs all exclude *INE-1*. A plus indicates that unique sequences flanking a HDR are in the collinear orientation in the *D. yakuba *genome. Orthologous regions could not be obtained for both flanking regions for HDRs at the tip or base of chromosome arms. Numbers of genes include coding and non-coding genes, with numbers of pseudogenes indicated in parentheses. *Likely to be fixed in *D. melanogaster*. ^†^Physical gap present in HDR. ^‡^HDRs 9 and 10 flank the *Histone *gene cluster and likely represent a single HDR. ^§^'Weak points' in polytene chromosomes.

**Table 3:** Patterns of nesting among different classes of TE in the *D. melanogaster *Release 4 genome sequence

	LTR	Non-LTR	TIR	*INE-1*	*FB*	Outer total
LTR	151	39	12	4	1	207
Non-LTR	46	46	10	17	0	119
TIR	28	29	32	18	6	113
*INE-1*	8	19	8	9	3	47
*FB*	0	4	0	1	0	5
Inner total	233	137	62	49	10	

Observed numbers of 25 possible categories of TE nests from 5 classes of TEs (LTR, non-LTR, TIR, *INE-1 *and *FB*) from 491 total nests.
